# Mesenchymal Stem Cells in Treatment of Spinal Cord Injury and Amyotrophic Lateral Sclerosis

**DOI:** 10.3389/fcell.2021.695900

**Published:** 2021-07-06

**Authors:** Eva Sykova, Dasa Cizkova, Sarka Kubinova

**Affiliations:** ^1^Institute of Neuroimmunology, Slovak Academy of Sciences, Bratislava, Slovakia; ^2^Centre for Experimental and Clinical Regenerative Medicine, University of Veterinary Medicine and Pharmacy in Kosice, Kosice, Slovakia; ^3^Department of Optical and Biophysical Systems, Institute of Physics of the Czech Academy of Sciences, Prague, Czechia

**Keywords:** mesenchymal stem cells, cell therapy, spinal cord injury, amyotrophic lateral sclerosis, neurodegenerative diseases, conditioned medium, exosomes, biomaterials

## Abstract

Preclinical and clinical studies with various stem cells, their secretomes, and extracellular vesicles (EVs) indicate their use as a promising strategy for the treatment of various diseases and tissue defects, including neurodegenerative diseases such as spinal cord injury (SCI) and amyotrophic lateral sclerosis (ALS). Autologous and allogenic mesenchymal stem cells (MSCs) are so far the best candidates for use in regenerative medicine. Here we review the effects of the implantation of MSCs (progenitors of mesodermal origin) in animal models of SCI and ALS and in clinical studies. MSCs possess multilineage differentiation potential and are easily expandable *in vitro*. These cells, obtained from bone marrow (BM), adipose tissue, Wharton jelly, or even other tissues, have immunomodulatory and paracrine potential, releasing a number of cytokines and factors which inhibit the proliferation of T cells, B cells, and natural killer cells and modify dendritic cell activity. They are hypoimmunogenic, migrate toward lesion sites, induce better regeneration, preserve perineuronal nets, and stimulate neural plasticity. There is a wide use of MSC systemic application or MSCs seeded on scaffolds and tissue bridges made from various synthetic and natural biomaterials, including human decellularized extracellular matrix (ECM) or nanofibers. The positive effects of MSC implantation have been recorded in animals with SCI lesions and ALS. Moreover, promising effects of autologous as well as allogenic MSCs for the treatment of SCI and ALS were demonstrated in recent clinical studies.

## Introduction

Adult stem cells and their secretomes play an important role in physiological conditions and in pathological states throughout our lives. Among adult stem cells, mesenchymal stem cells (MSCs) (Caplan, 1991) (progenitors of mesodermal origin) are of particular interest since they can be easily isolated from the bone marrow (BM), umbilical cord blood, umbilical Wharton jelly, and placenta. They can be separated, expanded *in vitro*, and implanted from autologous or allogenic sources. For these reasons, implantation of expanded MSCs or isolated secretomes or concentrated conditioned media from MSC cultivation can be used for treatment in animal models of degenerative diseases as well as in human clinical studies. Their beneficial effects on disease time course, accompanying symptoms, and life expectancy have been shown in neurodegenerative diseases such as Alzheimer’s disease (Nakano et al., 2020), spinal cord injury (SCI) (Pego et al., 2012; Liau et al., 2020), and amyotrophic lateral sclerosis (ALS) (Forostyak and Sykova, 2017; Barczewska et al., 2020).

In the last 10 years, the mechanism of MSC action has been gradually clarified. According to the International Society for Cellular Therapy position statement, MSCs are defined as cells which (1) adhere to plastic in culture conditions, (2) express CD105, CD73, and CD90 but not CD45, CD34, CD14, CD11b, CD79alpha, CD19, and HLA-DR surface molecules, (3) are able to differentiate *in vitro* into osteoblasts, adipocytes, and chondroblasts [see Dominici et al. (2006)]. Using specific procedures, MSCs have also been reported as being able to differentiate into neural cells and to express neuronal markers (Mezey et al., 2000b; Tropel et al., 2006; Park et al., 2012; Taran et al., 2014; Ullah et al., 2015). Although MSCs have the ability to differentiate into a variety of tissues, most of their effects are attributed to their paracrine action. MSCs produce and release a variety of biomolecules and soluble factors called secretomes. They are released from cells in the form of extracellular vesicles (EVs), i.e., lipid bilayer particles, which include microvesicles, apoptotic bodies, and exosomes. The proteomic analysis of MSC secretomes derived from the BM, adipose tissue, and fetal tissue has revealed trophic factors and cytokines as growth factors, immunomodulators, and antioxidants (Shin et al., 2021). Important growth factors are vascular endothelial growth factor (VEGF), basic fibroblast growth factor (bFGF), insulin-like growth factor (IGF-1), platelet-derived growth factor (PDGF), and hepatocyte growth factor (HGF). Anti-inflammatory factors such as interleukin 10 (IL-10) or transforming growth factor β1 (TGF-β1) have been shown to be involved in tissue repair and regeneration (Discher et al., 2009). MSC paracrine factors, therefore, have different functions, e.g., protecting against fibrosis, apoptosis, and oxidative damage, promoting angiogenesis, and conducting immunomodulatory and neuroprotective action.

Depending on the cellular microenvironment, MSCs can secrete neuroprotective growth factors in neural tissue, which can protect neurons and glial cells. Thus, secreted nerve growth factors like glia-derived nerve growth factor (GDNF), brain-derived growth factor (BDNF), VEGF, IGF-1, nerve growth factor (NGF), ciliary neurotrophic growth factor (CNTF), and neurotropin-3 (NT-3) promote different aspects of neural regeneration. Neurodegenerative diseases and traumatic brain or SCI caused by various pathologies and accidents often lead to permanent disabilities or death. MSCs and their secretomes are, therefore, accessible therapeutic tools for regenerative medicine, also including central nervous system (CNS) pathologies.

Damaged neural tissue is typically accompanied by cavitation induced by neural cell death, axonal degeneration, and tissue necrosis. This inevitably leads to scar formation, composed of inflammatory immune cells, fibroblasts, extracellular matrix (ECM) deposits, and astrocytes. MSCs have been shown to be anti-inflammatory, anti-apoptotic, and ECM modulatory (Marconi et al., 2012). Nevertheless, in chronic disease states, cavities and scars are impermeable barriers for tissue regeneration. For these reasons, tissue engineering and scaffold development often accompany cell therapies. Natural and synthetic scaffolds are developed to bridge the tissue defects. Moreover, various carriers for stem cell delivery and protection can enhance their effects.

In this review, we shall give examples of preclinical and clinical studies in SCI and motoneuronal disease or ALS (MND/ALS), focusing on the regenerative potential of MSCs, modulation of scar formation, ECM composition, and plasticity in CNS. We shall review recent development in scaffolds and tissue bridges. We are, of course, aware that MSC treatment can also be a useful strategy in the treatment of other neurodegenerative pathologies such as Alzheimer’s disease, stroke, or brain trauma.

## Spinal Cord Injury

Injury of the spine is a life-threatening neurodegenerative disorder leading to partial or complete loss of motor, sensory, and autonomic function below the injury. Spinal cord mechanical insult results in initial primary injury, when the spinal cord undergoes disruption caused by contusion, compression, transection, or stretching of the spinal column (Rosenzweig and McDonald, 2004; Ramer et al., 2005; Wang and Pearse, 2015). Secondary processes develop within minutes of the mechanical insult, manifested in subsequent progressive hemorrhaging, edema, thrombosis, ionic changes, ischemia, release of free radicals, lipid peroxidation, excitotoxicity, and apoptotic and necrotic cell death. All these together contribute to uncontrolled inflammation and immune response. It is necessary to understand that ongoing secondary mechanisms negatively affect the cells which survive within the primary injury site as well as those in the surrounding tissue, leading to enlargement of the lesion into adjacent spinal cord segments in rostro-caudal directions (Ramer et al., 2005). Moreover, progressing axonal degeneration together with tissue necrosis and cavity and scar formation ultimately preclude functional recovery (Silver and Miller, 2004; Forostyak et al., 2014; Bradbury and Burnside, 2019).

In this context, the diversity of secondary processes and the complexity of the spinal cord cyto-architecture together with its limited regenerative capacity in mammals are a major obstacle for finding an effective therapy (Sharif-Alhoseini et al., 2017). Better understanding of SCI pathophysiology, including genomic and proteomic profiles, may therefore provide opportunities for minimizing secondary pathological processes in adjacent healthy tissue (Ramer et al., 2005; Devaux et al., 2016).

### Conventional and Experimental Treatment

Patients with an injured spinal cord usually undergo standard neurosurgical procedures allowing for the safe decompression and stabilization of the spinal cord. This operational approach protects the nearest structures but has almost no impact on the progression of a secondary injury (Ahuja et al., 2017; Rath and Balain, 2017). Subsequently used pharmaceutical treatments are mainly aimed at suppressing a limited range of pathological processes of the same origin, for example, reducing inflammation and swelling, an approach which is neuroprotective but often ineffective overall and can leave patients paralyzed for the rest of their lives (Fawcett, 2009).

On the other hand, some positive effects have been achieved in SCI by combined neuro-protective–regenerative scaffold-based strategies such as therapeutic hypothermia, stem cells, biomaterials, and long-term targeted neuro-rehabilitation, all of which are being considered for experimental and clinical trials (Ahuja et al., 2017). Their multifactorial mechanisms of action may effectively protect injured tissue, enhance regeneration, and improve neurological function. Despite this possibility, however, traumatic SCI still results in severe or irreversible loss of function.

**FIGURE 1:**
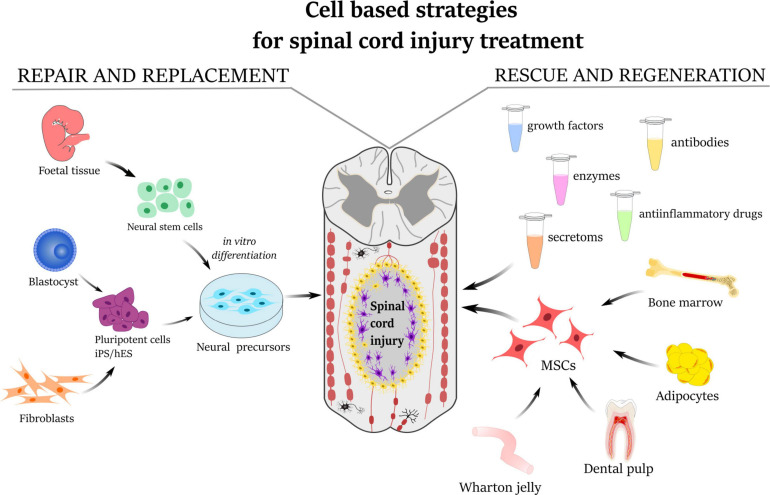
Schematic illustration describing the strategies for the treatment of spinal cord injury. Repair and replacement of damaged tissue by neural precursors derived either from fetal neural tissue or from induced pluripotent stem cells (iPS/hES). The mesenchymal stem cells derived from bone marrow, adipose tissue, dental pulp, or umbilical cord Wharton’s jelly have a rescue-and-regeneration effect mediated by paracrine action through releasing secretomes, growth factors, anti-inflammatory molecules as well as enzymes and antibodies.

Recently, studies using different types of stem cells [neural stem cells, induced pluripotent stem cells (iPS), and MSCs] or their conditioned media have been undergoing extensive research. One of the most commonly used therapies involves adult MSCs because MSCs are well recognized for releasing bioactive molecules, such as growth factors and cytokines, which have immunomodulatory, anti-inflammatory, anti-stress, angiogenic, and anti-apoptotic effects (Figure 1). However, the main shortcomings of MSC therapies lie in their unsatisfactory translation from small animal experimental models (mice and rats) into human clinical practice. It is necessary, therefore, to evaluate these therapies on animals which more closely resemble the anatomy of the spinal cord and immune response in humans and, at the same time, allow the performance of long-term follow-up studies (dogs, pigs, and primates) (Vikartovska et al., 2020). Moreover, it is important to bear in mind that no animal experimental model can completely match a human study.

### Stem Cell-Based Therapies

Strategies in regenerative medicine including cell-based nanotechnologies (Kubinová and Syková, 2010), spinal electric stimulation devices (Angeli et al., 2018; Gill et al., 2018), and targeted rehabilitation (Musselman et al., 2018) have shown progress in experimental and clinical therapies for CNS injuries (Kubinova and Sykova, 2012; Sykova and Forostyak, 2013). However, many years of research and practical experience have led to the conclusion that complex aspects need to be considered for successful treatment, for example, optimal timing/therapeutical window, delivery routes, the age and health condition of patients with SCI, and the use of a suitable source of stem cells or, alternatively, their EVs and their combination with advanced biomaterials (Grulova et al., 2015).

#### Bone Marrow Mesenchymal Stem Cells

Bone marrow mesenchymal stem cells (BMSCs) were the first cells used for traumatic injury treatment in experimental and clinical trials alike (Syková et al., 2006a). Primary studies based on the thoracic spinal cord contusion and BMSC transplantation in rats showed partial improvements in motor, sensory, and autonomic functions as well as in tissue sparing (Syková et al., 2006b). Interestingly, similar beneficial effects were detected when BMSCs were administered locally into the cavity of the spinal cord (Nandoe et al., 2006), intrathecally (Cizkova et al., 2011) or systemically (Cízková et al., 2006; Osaka et al., 2010). In a rat SCI model, as in balloon-induced compression lesion (Vanický et al., 2001), BMSCs were grafted intravenously at 1 week after injury. Behavioral testing revealed a significant improvement in motor and sensory tests (Jendelová et al., 2004).

**FIGURE 2:**
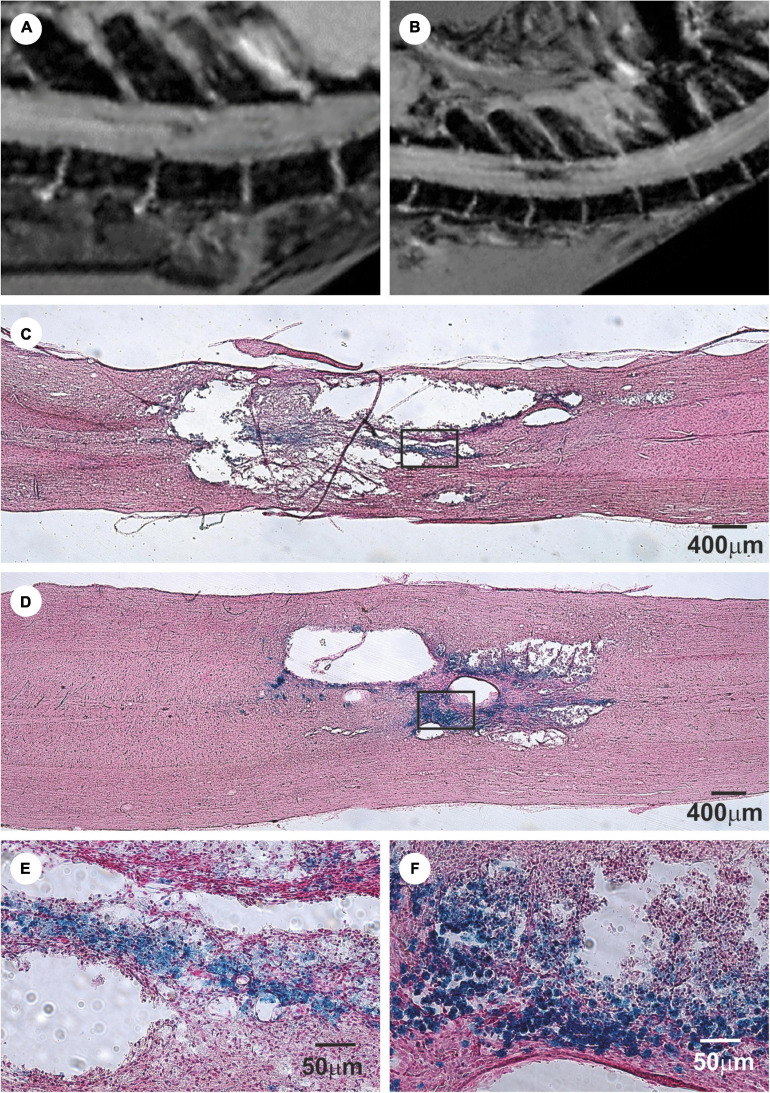
Bone marrow mesenchymal stem cells (BMSCs) labeled with iron-oxide nanoparticles implanted into rat with acute balloon-induced spinal cord compression lesion. **(A,B)** Longitudinal MRI images of spinal cord lesion. **(A)** At 5 weeks after compression the lesion was detected as a hyperintensive area with a weak hypointense signal. **(B)** Entire lesion populated by intravenously injected magnetically labeled BMSCs at 4 weeks after implantation is visible as a dark hypointensive area. **(C)** Prussian blue staining for iron of a spinal cord lesion in control animal. **(D)** Prussian blue staining for iron of a spinal cord lesion at 4 weeks after labeled BMSCs implantation. Note the smaller lesion size in the animal with implanted BMSC. **(E)** Prussian blue staining in detail shows a staining for hemoglobin. **(F)** The lesion is populated with Prussian blue-positive cells. Modified from Jendelová et al. (2004).

**FIGURE 3:**
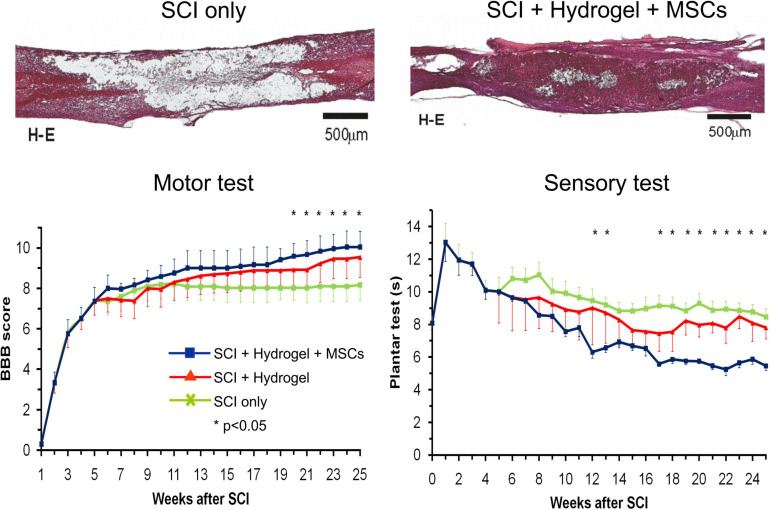
Hematoxylin–eosin staining of a rat spinal cord with acute balloon-induced spinal cord compression lesion. Spinal cord injury (SCI) only: longitudinal section of the spinal cord with a cavity in the control animal at 6 months after SCI. SCI + hydrogel + mesenchymal stem cells (MSCs): spinal cord at 6 months after a lesion with MSC-seeded HPMA-arginine–glycine–aspartic acid hydrogel implanted at 5 weeks after SCI. Motor test and sensory test: A comparison of motor test (Basso, Beattie, and Bresnahan score) and sensory test (plantar test) in 1–25 weeks after SCI in rats with SCI only, SCI and hydrogel implantation, and SCI with implantation of hydrogel seeded with bone marrow mesenchymal stem cells. Modified from Hejcl et al. (2010).

In these studies, magnetic resonance imaging (MRI) was used as a non-invasive method of studying the progress of transplanted cells in SCI *in vivo* (Jendelová et al., 2003, 2004; Syková and Jendelová, 2005). Superparamagnetic iron-oxide nanoparticles were inserted into adult BMSCs during their cultivation prior to their transplantation into the animals with SCI. The BMSCs were then visible in the MRI images of SCI as hypointensive signals persisting for more than 4 weeks (Figures 2A,B). *Ex vivo* Prussian blue histological staining for iron confirmed iron-positive cells at the lesion site (see Figures 2C–F) (Syková and Jendelová, 2005; Sykova and Jendelova, 2007). Chronic SCI is characterized by tissue loss (cavity formation and spinal atrophy), resulting in a stable functional deficit. It is therefore necessary to bridge any spinal cavity by implanting a functionalized scaffold (see section “Biomaterials In Combination With MSCs In SCI Treatment”). Partial recovery of motor and sensory function was found in chronic SCI after the implantation of hydrogel seeded *in vitro* with BMSCs (Figure 3) (Hejcl et al., 2010).

These promising preclinical studies initiated a series of I/II clinical trials delivering BMSCs (autologous and allogenic) or mononuclear fractions in patients with acute, sub-acute, or chronic SCI. In summary, the results from these clinical trials demonstrate that BMSCs are safe and without adverse effects. One of the first studies used all mononuclear cells from BM (Syková et al., 2006a). Partial improvement in the American Spinal Injury Association (ASIA) score and recovery of motor-evoked potentials and somato-sensory evoked potentials were observed in several patients when treated during the acute or sub-acute phase. Subsequently, a number of phase I/II clinical studies were launched in Korea, Japan, India, Egypt, China, Brazil, Chile, and Switzerland; an overview of these studies can be found on the clinicaltrial.gov website. The results of these studies are modest but promising. A comprehensive review of these studies has been published (Forostyak et al., 2013; Muthu et al., 2020; Silvestro et al., 2020), and it is beyond the scope of this review to list the results here. However, regardless of the promising results achieved, larger groups of patients are required before any practical statements can be drawn (Forostyak and Sykova, 2017).

#### Adipose Tissue Mesenchymal Stem Cells

Because BM isolation requires specialist intervention and there are certain limitations for donors, fat cells are presently being taken into account as well. They can be obtained more easily by means of liposuction or other surgical interventions (Vishnubalaji et al., 2012). Adipose tissue mesenchymal stem cells (ATMSCs) seem to have some similar characteristics with BMSCs, such as cell surface antigens expression, but they have different proliferation and multilineage capacities (Danisovic et al., 2009; Petrenko et al., 2020). Interestingly, contradictory data have been published in the relevant studies. Some studies indicate that ATMSCs are more effective than BMSCs, while others report that BMSCs are superior to ATMSCs (Elman et al., 2014).

Differences in the results can be obtained due to the fact that these cells differ in cytokine release, chemokine receptor expression, and apoptosis (Ahmadian Kia et al., 2011; Hsiao et al., 2012; Ruzicka et al., 2017). Furthermore, ATMSCs show a higher proliferative activity and are capable of secreting higher levels of IGF-1, VEGF-D, and interleukin-8 (Hsiao et al., 2012). In contrast, BMSCs are characterized by a slower proliferation but higher osteogenesis and chondrogenesis, and they secrete VEGF-A, angiogenin, bFGF, NGF, stem cell-derived factor-1, and HGF at comparable levels with ATMSCs (Ahmadian Kia et al., 2011). Due to these findings, ATMSCs tend to be preferred for stimulating angiogenesis. Thus, both types of stem cell have a range of biological activities and immunomodulatory properties which need to be considered when selecting these cells for a specific clinical trial (Huang et al., 2005; Zhou et al., 2020).

The ATMSC mechanisms underlying inflammatory suppression may be mediated by blocking the infiltration of ED1 macrophages as well as attenuating Notch1 signaling (Leu et al., 2010; Zhou et al., 2020). In a mouse model of SCI, the delivery of ATMSCs immediately after contusion led to decreased neuronal death and improvement in locomotion. Because transplanted ATMSCs do not differentiate into glial or neural cells, other processes may be responsible for this beneficial effect, such as the downstream factors of attenuated Notch1 signaling, including Jagged1, NICD, and RBP-JK (Zhou et al., 2020).

Follow-up clinical trials using intrathecal implantation of autologous ATMSCs in patients (*n* = 14) with SCI (at the cervical–thoracic and lumbar level) proved to be safe and revealed mild improvements in ASIA motor and sensory scores at 8 months of follow-up. Adverse events were observed in three patients, who suffered with urinary tract infection, headache, nausea, and vomiting (Hur et al., 2016). Similarly, a recently published case report from a phase 1 trial (CELLTOP study) declared that intrathecal autologous ATMSC delivery was feasible and safe with signs of an improved neurological condition (Bydon et al., 2020).

#### Umbilical Cord Wharton’s Jelly-Derived MSCs

Variability in experimental models of SCI and limited efficacy of adult stem cells may contribute to the final failure in clinical practice. A lot of effort has therefore been put into finding a more embryonic-like source of stem cells. Accordingly, the therapeutic potential of umbilical cord Wharton’s jelly-derived MSCs (WJMSCs) has emerged (Balasubramanian et al., 2013). These cells possess more embryonic-like properties with increased proliferation, reduced immunogenicity, and no tumorigenicity (Zhou et al., 2011; Kim et al., 2013). They secrete high levels of NGF, neurotrophic factors NT-3, NT-4, bFGF, and GDNF, and other molecules associated with neuroprotection, and they stimulate neurogenesis and angiogenesis (Balasubramanian et al., 2013). In a recent study, the repeated intrathecal delivery of WJMSCs into a rat ischemic–compression model of SCI showed the potentiated regeneration (Krupa et al., 2018) of the spinal cord in a dose-dependent manner. Histochemistry, in particular, indicated that higher doses of the delivered WJMSCs enhanced the number of GAP43-positive fibers, sparing the nerve tissue and reducing glial scar (Krupa et al., 2018).

#### MSC-Conditioned Media

As an alternative to cell-based therapies, conditioned media (CM) represent a cell-free product which can reduce undesirable immune issues, with effective mass production and storage and off-the-shelf availability. Similarly as with cell transplantations, the intrathecal administration of CM from BMSCs in a rat SCI model stimulated the intrinsic factors of spinal regeneration, resulting in tissue repair and motor function improvement (Kanekiyo et al., 2018). CM may be produced from various cells, such as BMSCs (Cizkova et al., 2018), dental pulp-derived MSCs (Asadi-Golshan et al., 2018), endothelial progenitor cells, or WJMSCs (Chudickova et al., 2019; Vawda et al., 2020). BMSC-CM contain anti-apoptotic, proinflammatory, neuromodulator, and angiogenic factors (Kanekiyo et al., 2018). When delivered for SCI treatment, they supported axonal regrowth and the recovery of locomotor function, reduced the lesion cavity, and promoted vascular stabilization (Cantinieaux et al., 2013). The content of CM has a limited concentration, so multiple injections over a longer time should be considered. CM may be delivered as intrathecal injections (Kanekiyo et al., 2018) or locally into the injured spinal cord tissue by means of an osmotic pump (Cantinieaux et al., 2013). Moreover, a systemic delivery of four intravenous injections of allogenic MSC-conditioned medium to dogs with chronic SCI proved to be safe and well tolerated (Vikartovska et al., 2020). MSC-CM in dogs, in combination with comprehensive and targeted physiotherapy, resulted in the improvement of the hind limb function and bladder control. This pilot study suggests that non-invasive, repeated injections of allogenic stem cell CM may substitute cell-based therapy and support spinal cord regeneration. However, to confirm the safety and efficacy of this treatment, it is necessary to involve a larger number of dogs and placebo controls during a long-term study (Vikartovska et al., 2020).

The most recently published report on high-throughput conditioned medium-secretome derived from umbilical cord matrix cells (HUCMCs) and BMSCs, as well as fibroblasts derived from newborn and adult tissue, compared their efficacy in a rat model of spinal clip compression injury. Data from this study indicate HUCMC-derived CM as being superior than the others tested due to the limitation of vascular pathology and participation in immune cell migratory pathways (MAPK/ERK, JAK/STAT) (Vawda et al., 2020).

#### Exosomes

Besides the growing interest in the beneficial effects of conditioned media on SCI, recent data highlight the therapeutic potential of cell-derived exosomes (Mendt et al., 2019). Exosomes are defined as small EVs composed of the lipid bilayers of a cell donor membrane, with a diameter of 50–150 nm (Théry et al., 2002). They are released through exocytosis by various cell types and can be detected in all body fluids (Murgoci et al., 2018). Interestingly, the intravenous delivery of MSC-derived exosomes in a rat model of SCI mitigated the severity of injury and enhanced functional recovery (Lankford et al., 2018). Most probably, MSC exosomes can mediate the transfer of miRNAs or release of trophic factors at the injury site and play a key role in intercellular communication (Li et al., 2018). Recently, it has been reported that MSC-derived exosomes migrated solely to the contused regions of the spinal cord and were associated with M2 macrophage-expressing CD206 (Lankford et al., 2018). Detailed analyses of exosomal content confirmed a complex cargo consisting of proteins, lipids, and short and long forms of RNA and DNA. The miR-133b found in MSC exosomes showed a therapeutic benefit in CNS trauma as well as in SCI (Li et al., 2018). The treatment of rats with miR-133b exosomes reduced spinal cavity volume, protected neuronal cells, and stimulated neurite outgrowth following SCI (Li et al., 2018). This may be attributed partially to the stimulation of ERK1/2, STAT3, and CREB and the attenuation of RhoA expression (Li et al., 2018). In a clinical study, miR-21 and miR-19b delivered by means of human MSC-derived EVs regulated the apoptosis and differentiation of neurons in patients with SCI (Xu et al., 2019). According to these findings, MSC-derived exosomes may treat SCI through angiogenic properties, stimulating axonal regeneration and suppressing the development of glial scar. These exosomes, similarly as the conditioned medium, present no risk of immune rejection, are more stable, and may be stored for a longer period than cells (Li et al., 2018).

Many years of studies using stem cells in regenerative medicine lead to the conclusion that the most efficient therapy for SCI can be based on a combination of biomaterial, stem cell, CM, or exosome therapies and molecule delivery (Xu et al., 2019). Despite the enormous scientific efforts being made so far in the research into SCI, the ultimate clarification of regeneration processes is still missing. However, care for SCI patients has significantly improved, and innovated surgical approaches together with supporting treatment and targeted rehabilitation can restore functionality to varying degrees and improve their quality of life.

## Biomaterials in Combination With MSCs in SCI Treatment

It is generally accepted that transplanted MSCs do not differentiate into neuronal or glial cells, but their therapeutic effects are associated with their ability to release a variety of antiapoptotic, neurotrophic, and anti-inflammatory molecules (Urdzikova et al., 2014; Ruzicka et al., 2017; Krupa et al., 2018; Chudickova et al., 2019; Petrenko et al., 2020). In line with this concept, we and others have previously demonstrated the regenerative effects and functional recovery in SCI after intrathecal MSC transplantation, without the transplanted cells being integrated into the damaged tissue (Urdzikova et al., 2014; Krupa et al., 2018). Moreover, a comparable therapeutic effect has also been shown after the intrathecal delivery of conditioned media containing a complex of MSC-secreted products, developed as a cell-free alternative to cell therapy (Amemori et al., 2015; Cizkova et al., 2018; Chudickova et al., 2019). In contrast to intrathecal application, intralesional cell transplantation may provide higher cell retention and more localized and focused cell effects at the site of delivery. However, the efficacy of intralesional transplantation is often limited by poor cell survival in the unfavorable microenvironment of the injured neural tissue.

**FIGURE 4:**
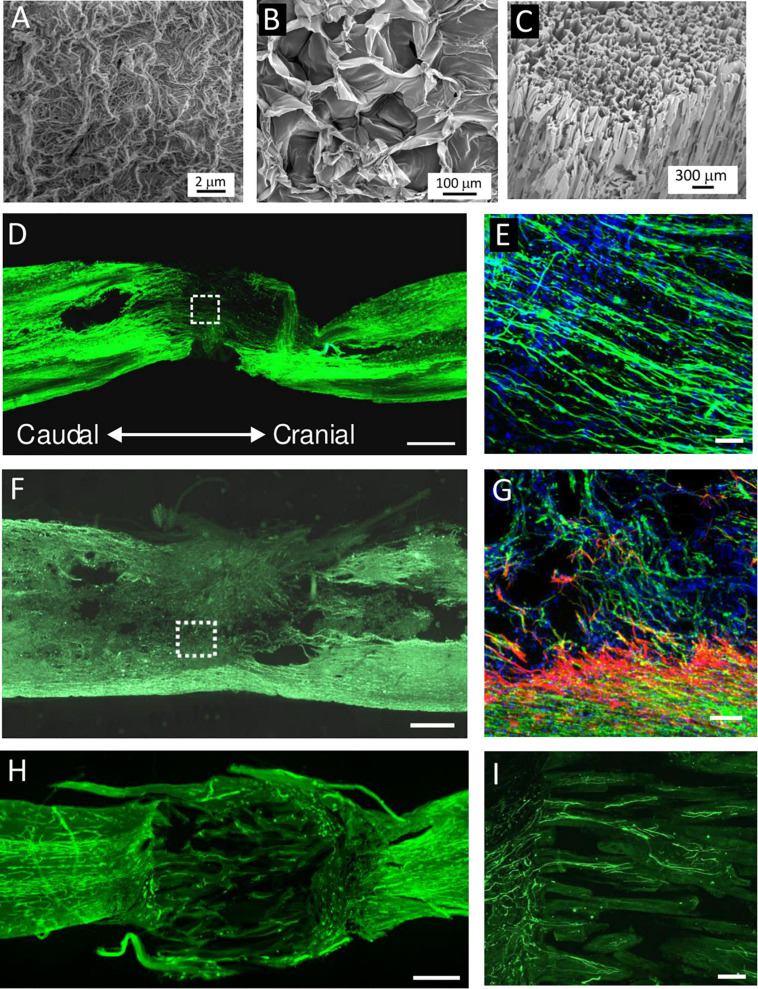
**(A–C)** SEM micrographs of **(A)** extracellular matrix (ECM) hydrogel, **(B)** hyaluronic acid (HA) hydrogel modified with arginine–glycine–aspartic acid (RGD), and **(C)** highly superporous SIKVAV-modified superporous poly (2-hydroxyethyl methacrylate) hydrogel scaffolds with oriented pores. **(D–I)** Representative images of the longitudinal sections of the spinal cord lesion after hydrogel injection or implantation into the hemisection cavity. **(D,E)** Immunofluorescence staining for neurofilaments (NF-160, green) and **(E)** cell nuclei (DAPI, blue) at 2 weeks after the injection of ECM hydrogel derived from porcine spinal cord. **(F,G)** Immunofluorescence staining for neurofilaments (NF-160, green), **(G)** astrocytes (GFAP, red), and cell nuclei (DAPI, blue) at 8 weeks after HA–RGD hydrogel implantation; the square in panel **(F)** is shown under the higher magnification inset in panel **(G)**. **(H,I)** Immunofluorescence staining for **(H)** blood vessels (RECA) and **(I)** neurofilaments (NF-160, green) at 2 months after the implantation of SIKVAV-modified superporous poly (2-hydroxyethyl methacrylate) hydrogel with parallel-oriented pores. Scale bar: **(D,F,H)** 500 μmm, **(E,G)** 50 μmm, and **(I)** 100 μmm. Modified from **(A)** Koci et al. (2017), **(D,E)** Tukmachev et al. (2016), **(B,F,G)** Zaviskova et al. (2018), and **(C,H,I)** Kubinova et al. (2015).

Various biomaterials of synthetic [e.g., 2-hydroxyethylmethacrylate, hydroxypropylmethacrylamid, poly L-lactic acid, poly(lactic-co-glycolic acid), poly-L-lysine, and polyethylene glycol] as well as natural origin [e.g., Hyaluronic acid (HA), alginate, chitosan, collagen, fibrin, and ECM scaffolds] have been developed to bridge the lesion and provide a stimulatory microenvironment to support the survival and efficacy of transplanted cells (Figures 3, 4) (Hejcl et al., 2010; Kubinova and Sykova, 2012; Liu et al., 2019). In fact, scaffolds promote MSC adhesion and their survival. The effects of cell-seeded biomaterial scaffolds on improved axonal regrowth or enhanced functional outcomes after SCI than scaffolds alone have been shown in many studies (Hejcl et al., 2010; Kim et al., 2016; Blasko et al., 2017; Peng et al., 2018; Zaviskova et al., 2018) and reviewed in Libro et al. (2017) and Yousefifard et al. (2019).

The composition of scaffolds together with their physical properties, such as stiffness, pore size, and porosity, or three-dimensional structures should mimic the target tissue and allow appropriate scaffold integration into the site of transplantation. Additionally, the development of different types of scaffolds for 3D culture enables the generation of *in vitro* neural-like tissue as a new approach for modeling and tackling diseases of the brain and CNS (Murphy et al., 2017).

### Hydrogels

Hydrogels have proved to be the most convenient material, especially due to their ability to retain high levels of water and potential to adjust their mechanical properties to imitate soft nervous tissue. The benefit of the hydrogels is their injectability, which enables *in situ* gelation in lesion cavities of irregular shape together with cell or drug encapsulation. Hydrogels for CNS repair are commonly based on ECM, such as HA, collagen, or gelatine. HA, as a component of the ECM, is a biomaterial which is widely used in various clinical settings; it is biocompatible, biodegradable, and non-immunogenic. Native HA does not form a gel or promote cell adhesion, so various physical and chemical crosslinking methods have been developed to prepare injectable HA hydrogels. We previously demonstrated the neuroregenerative potential of an enzymatically crosslinked hydroxyphenyl derivative of HA modified with the integrin-binding peptide arginine–glycine–aspartic acid in the case of subacute spinal cord hemisection (Zaviskova et al., 2018). The hydrogels filled the lesion, promoting vascularization and axonal ingrowth into the lesion, and this effect was further potentiated in combination with human Wharton’s jelly-derived MSCs (Zaviskova et al., 2018).

### Extracellular Matrix Scaffolds

Other interesting materials are biological ECM scaffolds prepared by means of tissue decellularization, which removes cellular components from the tissue. These materials recapitulate the complex biochemical composition of ECM and mimic the native cell environment. The composition of ECM scaffolds can vary between tissues, but the most abundant compounds are collagen, glycosaminoglycans, fibronectin, or laminin (Costa et al., 2017). Generally speaking, decellularized scaffolds can be prepared as solid fibrous structures, sponges, or sheets and can also be further solubilized into the form of injectable ECM hydrogel (Costa et al., 2017). The advantage of ECM hydrogels is their ability to physically crosslink *in situ* at physiological pH and temperature, which allows their non-invasive injection into the lesion or cavity (Kubinova, 2017).

For CNS repair, ECM hydrogels derived from porcine brain, spinal cord, or urinary bladder has been evaluated in *in vitro* as well as *in vivo* studies (Crapo et al., 2014; Hong et al., 2020). We demonstrated that ECM hydrogels derived from decellularized porcine spinal cord and urinary bladder tissues filled the lesion, had an effect on the immune response, and created a stimulatory substrate for *in vivo* neural tissue repair after SCI. On the other hand, no significant changes were found in chemotactic or neurotrophic properties *in vitro* or *in vivo* between CNS-derived and non-CNS-derived ECM hydrogels, which do not indicate any detectable tissue-specific effect of the neural ECM (Tukmachev et al., 2016). Remarkably, using adult CNS tissues as a source of ECM matrix might be limited due to the presence of factors which suppress axonal growth, such as chondroitin sulfate proteoglycans and myelin-associated molecules. In this context, it has been demonstrated that decellularization in the adult optic nerve selectively removes the inhibitory compounds of the CNS tissue and preserves some axon-promoting ECM proteins, including collagen IV and laminin (Sun et al., 2020). It should therefore be emphasized that, for the development of CNS-derived ECM scaffolds, the extent of tissue decellularization must maintain an optimal balance between the effective clearance of myelin and myelin-related inhibitory factors while retaining compounds with neurotrophic properties.

Besides xenogeneic or allogeneic cadaveric tissues, ECM derived from fetal human tissue, such as the umbilical cord, represents a promising source for tissue engineering due to its human origin, easy accessibility, and absence of ethical constraints. We developed efficient and reproducible decellularization protocols for the production of ECM-based hydrogel derived from human umbilical cord tissue and proved its *in vitro* biocompatibility and similarity with ECMs derived from porcine tissues such as urinary bladder, spinal cord, and brain (Tukmachev et al., 2016; Koci et al., 2017). Moreover, the mechanical strength and bio-stability of ECM hydrogels can be further improved by crosslinking with genipin (Vyborny et al., 2019).

### Conduits for Axonal Guiding in SCI Repair

Injectable hydrogels are suitable carriers for cell or drug delivery, but during *in situ* gelation, they usually do not allow controllable microporosity, which could guide regenerating axon growth through the lesion. For this purpose, various types of solid fibrous or multichannel polymer conduits have been proposed as providing directional support for regrowing axons together with cell or drug delivery (Liu et al., 2017). Such conduits are mostly implanted into the complete spinal cord transection after removal of the spinal segment. Importantly, micro/nano-structures in nerve conduits have proved to be essential in tuning a large variety of post-implantation effects (Sun et al., 2019).

For example, Chen et al. (2020b) showed the regenerative effect of BMSCs seeded into a chitosan tubular scaffold combining the two architectures of a single H-shaped central tube and several microchannels. The scaffold was implanted to bridge the 5-mm defect of a complete transverse lesion in the thoracic spinal cord of rats, and when compared with the empty scaffold, the BMSC enhanced functional improvement and the number of regenerating axons and elicited antiapoptotic effects (Chen et al., 2020b). Peng et al. (2018) showed that rat MSCs combined with a nerve-guide collagen scaffold inhibited chronic scar formation, provided linear guidance for the nerves, and promoted M2 polarization to form an anti-inflammatory environment in a hemisected SCI rat model (Peng et al., 2018). Deng et al. (2020) transplanted human WJMSCs on collagen scaffolds into complete spinal cord transection in rats and dogs. The transplantation improved motor scores, enhanced amplitude, shortened the latency of motor evoked potential, and decreased the lesion area, which was further potentiated when the scaffold was used in combination with stem cells (Deng et al., 2020).

We previously tested SIKVAV-modified superporous poly (2-hydroxyethyl methacrylate) hydrogel with oriented pores (Kubinova et al., 2015). The hydrogels, either empty or seeded with rat MSCs, were implanted in the spinal cord transection. However, MSCs seeded in the scaffold did not enhance tissue infiltration into the pores, and only rare axons crossing the hydrogel bridge were observed after 6 months, which suggests that this type of scaffold did not provide an optimal environment for neural tissue repair (Hejcl et al., 2018).

To support the effects of MSCs, combined strategies have been proposed to further stimulate axonal growth and tissue regeneration. For example, the effect of rat BM-MSCs in a multichannel polymer poly (lactic-co-glycolic acid) scaffold was enhanced by their co-transplantation with Schwann cells, which promoted MSC survival and differentiation into neuron-like cells and resulted in the regeneration of axons and functional recovery after complete spinal cord transection (Yang et al., 2017).

### Clinical Trials Using Scaffolds in SCI Repair

Despite the number of preclinical studies using various scaffolds for SCI repair, only a few of them are currently approved for clinical trials. The safety and benefit of implantation of poly(lactic-co-glycolic acid)-b-poly(L-lysine) scaffold (neuro-spinal scaffold) has been evaluated in patients with thoracic AISA A spinal cord injury at a level of injury of T2–T12 (NCT02138110). No adverse effects related to acute scaffold implantation were reported in the 6-month study (Layer et al., 2017).

In another clinical study, a collagen scaffold with linearly aligned pores and functionalized with neuroactive factors (NeuroRegen scaffold) was loaded with autologous BM mononuclear cells and transplanted into the surgically cleaned lesion in seven patients with acute complete SCI. No adverse symptoms were present in the 3-year follow-up period. In some patients, partial sensory and autonomic nervous functional improvements, but no motor function recovery, were observed (Chen et al., 2020a). In the following study, a NeuroRegen scaffold was loaded with human WJMSCs and implanted into the surgically cleaned lesion in eight patients with chronic complete SCI. No adverse events were reported during 1 year of follow-up. In some patients, increase of sensation level and motor evoked potential-responsive area, enhanced finger activity and trunk stability, defecation sensation, and autonomic neural function recovery were found (Zhao et al., 2017).

Deng et al. (2020) published the results of a phase I clinical trial on 40 patients with acute complete cervical injuries. The group of patients (*n* = 20) obtained collagen scaffolds seeded with MSCs derived from umbilical cord tissue. No serious complications were reported during the 12-month follow-up. In the treatment group, an improvement in neurological functions was observed over the follow-up period, while no neurological functions were improved in the control group of patients (Deng et al., 2020).

Encouraging first clinical results indicate the safety and feasibility of MSC-seeded scaffold-based therapy in SCI repair; however, the observed weak functional recovery suggests the need to develop more advanced combinatorial approaches which would further target the inhibitory environment of the adult CNS tissue as well as the limited regenerative ability of the long-track axons.

## Mesenchymal Stromal Cells for the Treatment of ALS

Amyotrophic lateral sclerosis or MND is a devastating, rapidly progressing, and fatal neurodegenerative disease which attacks motoneurons (MNs) in the anterior horn of the spinal cord. Patients exhibit atrophy of the spinal cord and often also atrophy of cerebral gray and white matter. The disease is characterized by muscle weakness and atrophy, fasciculations, spasticity, and paralysis, which are leading to death usually within 3–5 years after the onset of clinical symptoms. Some patients exhibit a slower time course of the disease. About 90% of all cases are sporadic, while 10% of patients suffer from a familial disease. Researchers and clinicians worldwide have been searching for an effective treatment of this devastating disease for many years. Poor prognosis and symptomatic treatment are so far the only prospect for patients. The pharmaceutical treatments used in all patients include glutamate inhibition with riluzole (Bensimon et al., 1994), which only extends survival by about 3 months, or free radical scavenger edaravone (Cho and Shukla, 2020). Both of these drugs only delay the symptomatic and pathological progression of ALS. Apart from this, patients can get some relief from secondary complications through neurorehabilitation.

Stem cell-based therapies are potentially effective treatments for ALS patients (Forostyak and Sykova, 2017; Goutman et al., 2019). There are generally two strategies in using stem cells: firstly, achieving the replacement of lost motoneurons or pathological astrocytes, in particular, using a ready source of induced pluripotent stem cells (iPSCs) (Sareen et al., 2014; Goutman et al., 2019) and, secondly, using adult stem cells which can play a supportive role and provide a neuroprotective environment. This purpose may be served by autologous or allogenic undifferentiated MSCs of various origin, derived from the BM, umbilical cord blood, adipose tissue, or Wharton’s jelly. MSCs from different sources show some differences in growth rate, molecular phenotype, cell marker expression, and ability to differentiate into neuronal- or glial-like phenotypes (Blondheim et al., 2006; Zhu et al., 2008; Forostyak et al., 2016b), but they generally share some common features: they grow extensively in culture and differentiate *in vitro* into chondrocytes, osteocytes, adipocytes, and muscle cells, and they can be obtained for autologous application (Prockop, 1997; Mezey et al., 2000a; Krause, 2002; Forostyak et al., 2016a).

Mesenchymal stem cells of a different origin have been tested in rodent models to treat diseases such as ALS. Numerous preclinical studies with mutant superoxide dismutase 1 (SOD1) in mouse or rat have been performed. These preclinical studies have demonstrated that the intrathecal, intraspinal, intravenous, or combined intraspinal and intravenous administration of MSCs is a safe procedure which is able to slow down motor impairment, decrease inflammation, and stimulate the secretion of specific cytokines and growth factors which promote cell survival and enable symptomatic transgenic animals to survive longer. The single or repeated transplantation of MSCs induces the secretion of BDNF, VEGF, NGF, GDNF, and IGF-1, which play a crucial role in neuroregeneration (Uccelli et al., 2011; Li et al., 2002; Zhang et al., 2004; Vercelli et al., 2008; Gu et al., 2009). Their paracrine action rather than cell replacement supports the resistance of neurons and glia to apoptosis due to the release of anti-apoptotic and trophic factors, thus maintaining a neuroprotective microenvironment.

We found that engraftment of human MSCs into symptomatic ALS rats was able to preserve MNs. It decreased the extent of apoptosis in motor neurons, supported the survival of larger-sized neurons, and modified the affected ECM and cytokine homeostasis (Forostyak et al., 2011, 2014). The MSCs in these animals had anti-inflammatory and neuroprotective effects, and due to their ability to remodel the gene expression profile of the recipient, they activated CNS plasticity. *Wisteria floribunda* agglutinin (WFA) fluorescence intensity, measured in the ventral horns of the cervical and lumbar spinal cord, revealed greater numbers of perineuronal nets (PNNs) in the MSC-treated animals when compared with the control group. In our preclinical study, the MSCs were delivered intrathecally into symptomatic SOD1 G93A transgenic rats, and survival in the MSC-treated group was prolonged by 13.6 days compared with the control group. The cell-treated rats showed better motility and grip strength test results; there were significantly greater numbers of motoneurons compared to non-treated animals and less apoptotic activity (TUNEL assay). Applying quantitative analyses of WFA fluorescence intensity, we found preserved PNNs (Forostyak et al., 2011, 2014). PNNs have been shown to affect CNS plasticity and to protect neurons during injury and neurodegeneration (Sorg et al., 2016; Fawcett et al., 2019; Chelyshev et al., 2020; Yang, 2020). Moreover, the concomitant intraspinal and intravenous transplantation of rat MSCs resulted in neuroprotective effects also due to decreased inflammation, suppressed proliferation of microglial cells, and reduced expression of COX-2 and NOX-2, which increased motor activity and extended the lifespan of ALS rats (Boucherie et al., 2009; Forostyak et al., 2011).

A similar positive effect on motor activity and longer animal survival was found after the intravenous application of human umbilical cord blood and MSC in asymptomatic rat models of ALS (Mazzini et al., 2004; Garbuzova-Davis et al., 2008; Vercelli et al., 2008; Kim et al., 2010).

### MSCs in Clinical Trials for ALS

There is a spectrum of stem cells which can be considered for human clinical trials in neurodegenerative diseases. In ALS BM mononuclear cells, olfactory ensheathing cells, iPSCs, and fetal neural precursor cells have been used. An overview of the clinical studies in ALS can be found on the clinicaltrial.gov website. Here we review human ALS clinical trials using MSCs. It is evident that the number of phase I/II clinical trials is increasing annually. The majority of approved clinical trials employ autologous MSCs derived from BM. They can be easily obtained by BM aspiration and then expanded *ex vivo*. This is a minimally invasive procedure, and manipulation with autologous cells from patients, legal issues, and the long history of clinical application of BM-derived cells make them ideal candidates for stem cell therapy. These cells are quite unique, especially for their paracrine properties. Autologous MSC application does not require any immunosuppression, and there is no evidence of malignant transformation (Forostyak and Sykova, 2017). MSC implantation in animal models has revealed the paracrine production of growth factors and cytokines (see above). Furthermore, since CNS neuroinflammation plays an important role in neurodegenerative diseases, the anti-inflammatory influence of MSC can also explain their beneficial effect in clinical trials. Similarly as in SCI, different methods of MSCs and stem cell delivery have been used (Forostyak et al., 2014).

Successful experiments with rodent models of ALS have established a platform for clinical trials involving patients (Vercelli et al., 2008). Current and future clinical trials using stem cells for ALS treatment have been summarized in several reviews (Forostyak and Sykova, 2017; Goutman et al., 2019; Sharma et al., 2019). These clinical trials are mostly safety studies involving small numbers of patients. Majority of these trials do not present enough details about the types of cell used, dosage of stem cells, and criteria for patient monitoring or do not sufficiently report the study outcomes. Proper interpretation of the data is impossible, thus complicating its further clinical application. Most of the trials performed so far did not include patient follow-up for longer than 24 months. The first long-term outcome was studied after 5 years of monitoring 19 ALS patients treated with MSCs. These patients were enrolled in two phase I clinical trials, but no clear clinical benefits of MSC implantation was found. However, the collected data show support for the implantation of autologous BMSCs into the spinal cord, as no structural changes, tumor formation, or deterioration in psychosocial status were found, and all patients coped well with the procedure (Mazzini et al., 2003, 2010, 2011). Another study injected a mononuclear CD133(+) fraction from autologous stem cells isolated from the peripheral blood and into the frontal motor cortex of ALS patients (Martinez et al., 2009). This application of mononuclear cells prolonged the survival of the treated patients and increased the quality of their life compared with the control (untreated patients). Deda et al. (2009) reported the results of a 1-year follow-up of patients with the implantation of BM-derived hematopoietic progenitor stem cells into the anterior part of the spinal cord. From 13 patients with a bulbar form of ALS, nine patients became much better compared with their pre-operative status; one patient was stable, without any decline or improvement in his status, and three patients died at 1.5, 2, and 9 months after the stem cell therapy due to lung infection and myocardial infarction (Deda et al., 2009). Petrou et al. (2016) performed a safety study with MSCs secreting a neurotrophic factor (Petrou et al., 2016).

**FIGURE 5:**
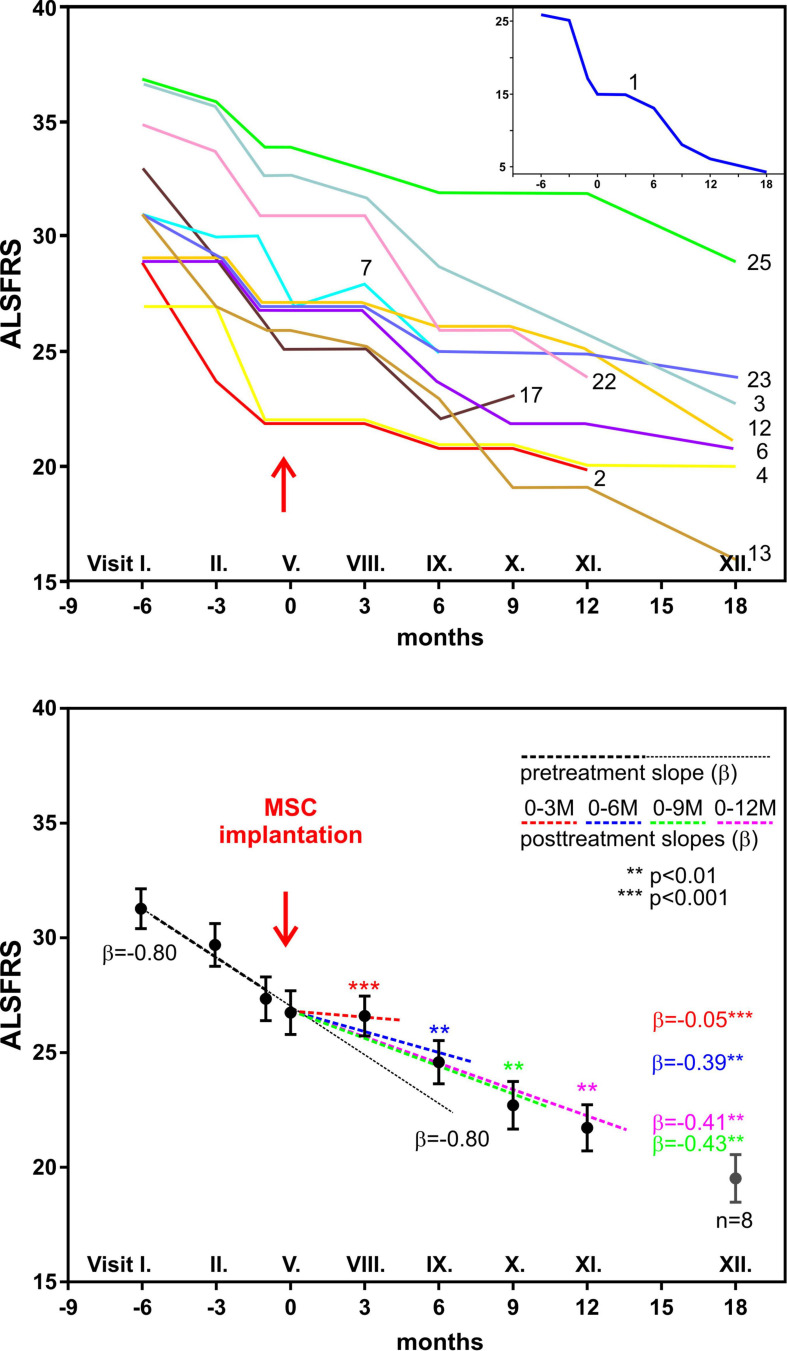
Clinical analysis of 12 amyotrophic lateral sclerosis patients with fast decline of functional rating scale (ALSFRS) scores 6 months before and 12 months after autologous bone marrow mesenchymal stem cell (BMSC) application. The upper panel shows the time courses of ALSFRS scores in individual patients; patient no. 1 is shown in the inset. The lower panel shows the regression analysis of ALSFRS scores before and after BMSC application. The solid line with β = –0.80 is the predicted time course without BMSC treatment. Modified from Sykova et al. (2017).

Our prospective, non-randomized, open-label clinical trial has been performed in Prague, Czechia (Sykova et al., 2017). This study concentrated on the safety and efficacy assessment of autologous multipotent MSC application in the treatment of patients with a confirmed diagnosis of ALS. The trial involved 26 patients with sporadic ALS, who received a single intrathecal dose of autologous MSCs applied into the cerebrospinal fluid. The intrathecal application seems to be preferable than intravenous administration, where the cells can be trapped in different organs. Intrathecally implanted cells immediately spread in the CSF around the brain and spinal cord, without the need to cross the blood–brain barrier. Compared to previous MSC trials, this study included the largest group of ALS patients and had a longer pre- and post-treatment assessment period, and only a single dose of stem cells was used. In the 18-month follow-up period, potential adverse reactions were assessed by means of clinical, laboratory, and MRI examination. The clinical outcome was evaluated using ALS functional rating scale (ALSFRS), Norris spinal and bulbar scale, forced vital capacity (FVC), and weakness scale. After MSC application, 30% of patients experienced mild/moderate headache, typically observed as resembling the headache after a standard lumbar puncture. No suspected serious adverse reactions or a cerebrospinal pathology was found during the MRI examinations. In almost 80% of patients, the FVC values remained above 60% for a time period of 12 months. In a group of 12 patients with a remarkable pretreatment decline in functional scales, we found a significant mitigation/stabilization in their total functional score decline at 3 months after application, which was less pronounced at 6 and 9 months (Figure 5).

Another small study using autologous BMSCs applied either intrathecally or intravenously similarly showed a slower deterioration in ALSFRS-R score, with the FVC remaining stable for about 6 months, and longer survival (Karussis et al., 2010; Prabhakar et al., 2012; Oh et al., 2015; Rushkevich et al., 2015; Sharma et al., 2015). Repeated intrathecal MSC application with similar positive and longer-lasting effects was reported by Oh et al. (2015, 2018). It is an important finding that some studies report a better and longer-lasting outcome after repeated applications of MSCs. A recent and larger study by Barczewska et al. (2020) used umbilical cord MSCs in a case–control study involving 67 patients (Barczewska et al., 2020). The patients were treated with WJMSCs, with three intrathecal injections every 2 months at a dose of 30 × 10^6^ cells. The authors report that median survival time increased twofold in all patients, and in some patients, there was a decrease in progression rate. Sharma et al. (2020) found in a case study that cell therapy, along with intensive physical rehabilitation, significantly improved the outcomes in a 40-year-old male ALS patient suffering for the preceding 4 years and who underwent multiple doses of cell therapy (Sharma et al., 2020).

## Conclusion

To conclude, even though all the above-mentioned studies report similar outcomes, thus corroborating the safety of the procedure, there is a need for more extensive multicenter trials. Even though a small series of experiments involving patients suggests an improvement in motor and sensory functions after the administration of MSCs, significant obstacles remain before these findings can be translated into novel therapies. In particular, we need to better understand the mechanisms of MSC action and the behavior of the transplanted stem cells in the pathological environment of CNS. More clinical trials with larger and more homogeneous groups of patients and with a longer follow-up are needed to enable better evaluation of stem cells treatments (Lindvall and Kokaia, 2006). It is also necessary to recall the fact that neuroprotective effects after cell-based therapy have been achieved in trials employing different routes of application, so the combination of different methods of cell delivery might produce even better results related to survival and motor functions. Finally, the development of specific markers enabling an early disease diagnosis will be of great importance for the evaluation of a possible effect of cell-based therapy. It is particularly important because, at the beginning of neurodegeneration, stem cells might produce more benefits in rescuing neurons from death, influence changes in ECM, and stimulate plasticity.

## Author Contributions

All authors listed have made a substantial, direct and intellectual contribution to the work, and approved it for publication.

## Conflict of Interest

The authors declare that the research was conducted in the absence of any commercial or financial relationships that could be construed as a potential conflict of interest.
