# Corrigendum: Mesenchymal Stem Cells in Treatment of Spinal Cord Injury and Amyotrophic Lateral Sclerosis

**DOI:** 10.3389/fcell.2021.770243

**Published:** 2021-10-28

**Authors:** Eva Sykova, Dasa Cizkova, Sarka Kubinova

**Affiliations:** ^1^Institute of Neuroimmunology, Slovak Academy of Sciences, Bratislava, Slovakia; ^2^Centre for Experimental and Clinical Regenerative Medicine, University of Veterinary Medicine and Pharmacy in Kosice, Kosice, Slovakia; ^3^Department of Optical and Biophysical Systems, Institute of Physics of the Czech Academy of Sciences, Prague, Czechia

**Keywords:** mesenchymal stem cells, cell therapy, spinal cord injury, amyotrophic lateral sclerosis, neurodegenerative diseases, conditioned medium, exosomes, biomaterials

In the original article, there was a mistake in the caption of **Figure 2** as published. The description of part (A) was incorrect. The corrected caption appears below.

**Figure 2 |** Bone marrow mesenchymal stem cells (BMSCs) labeled with iron-oxide nanoparticles implanted into rat with acute balloon-induced spinal cord compression lesion. **(A,B)** Longitudinal MRI images of spinal cord lesion. **(A)** At 5 weeks after compression the lesion was detected as a hyperintensive area with a weak hypointense signal. **(B)** Entire lesion populated by intravenously injected magnetically labeled BMSCs at 4 weeks after implantation is visible as a dark hypointensive area. **(C)** Prussian blue staining for iron of a spinal cord lesion in control animal. **(D)** Prussian blue staining for iron of a spinal cord lesion at 4 weeks after labeled BMSCs implantation. Note the smaller lesion size in the animal with implanted BMSC. **(E)** Prussian blue staining in detail shows a staining for hemoglobin. **(F)** The lesion is populated with Prussian blue-positive cells. Modified from Jendelová et al. (2004).

The authors apologize for this error and state that this does not change the scientific conclusions of the article in any way. The original article has been updated.

## Publisher's Note

All claims expressed in this article are solely those of the authors and do not necessarily represent those of their affiliated organizations, or those of the publisher, the editors and the reviewers. Any product that may be evaluated in this article, or claim that may be made by its manufacturer, is not guaranteed or endorsed by the publisher.
